# Consumer Perspectives on the Adoption of a Prehabilitation Multimodal Online Program for Patients Undergoing Cancer Surgery

**DOI:** 10.3390/cancers15205039

**Published:** 2023-10-18

**Authors:** Daniel Steffens, Linda Denehy, Michael Solomon, Cherry Koh, Nabila Ansari, Kate McBride, Sharon Carey, Jenna Bartyn, Aaron Sean Lawrence, Kym Sheehan, Kim Delbaere

**Affiliations:** 1 Surgical Outcomes Research Centre (SOuRCe), Royal Prince Alfred Hospital (RPAH), Sydney, NSW 2050, Australia; professor.solomon@sydney.edu.au (M.S.); cherry_koh@hotmail.com (C.K.); drnabilaansari@gmail.com (N.A.); kate.mcbride@health.nsw.gov.au (K.M.); sharon.carey1@health.nsw.gov.au (S.C.); jenna.bartyn@health.nsw.gov.au (J.B.); alaw0415@uni.sydney.edu.au (A.S.L.); kymshe64@gmail.com (K.S.); 2 Faculty of Medicine and Health, Central Clinical School, The University of Sydney, Sydney, NSW 2050, Australia; 3 Institute of Academic Surgery (IAS), Royal Prince Alfred Hospital (RPAH), Sydney, NSW 2050, Australia; 4 Department of Physiotherapy, Faculty of Medicine Dentistry and Health Sciences, The University of Melbourne, Melbourne, VIC 3010, Australia; l.denehy@unimelb.edu.au; 5 Department of Health Services Research: Allied Health, Peter MacCallum Cancer Centre, Melbourne 3052, Australia; 6 Colorectal Department, Royal Prince Alfred Hospital (RPAH), Sydney, NSW 2050, Australia; 7 Nutrition and Dietetics Department, Royal Prince Alfred Hospital (RPAH), Sydney, NSW 2050, Australia; 8 Neuroscience Research Australia, Sydney, NSW 2031, Australia; k.delbaere@neura.edu.au; 9 School of Population Health, University of New South Wales, Kensington, NSW 2052, Australia

**Keywords:** prehabilitation, consumer, application, cancer, surgery, exercise, nutrition, psychological

## Abstract

**Simple Summary:**

This study explored the views of 30 gastrointestinal cancer patients on the adoption of a prehabilitation multimodal program, including exercise, nutrition and psychological counselling, which was delivered via e-Health. Most of the patients undergoing gastrointestinal cancer surgery were confident using technology, perceived the preoperative online program safe to be delivered at home, and of potential benefit to their wellbeing. However, “poor preoperative health”, “lack of motivation” and “lack of personal encouragement” were identified as the main barriers to the uptake of a preoperative online program. The safety and efficacy of the online program would need to be tested in a larger randomised controlled trial.

**Abstract:**

This study aimed to explore patients’ perspectives on the adoption of a prehabilitation multimodal online program. Patients recovering from gastrointestinal cancer surgery at a tertiary hospital between October 2021 and November 2022 were invited to participate. An e-Health program including intensity exercises, nutrition and psychological counselling was used. Patients were instructed to navigate the e-Health program over 24 h using an iPad and then complete the study survey. Patients’ characteristics, use of technology, views and minimal expected outcomes from a preoperative online program were collected. Of the 30 patients included, most were female, most reported confidence in the use of technology, most considered the online program safe and most agreed it would be beneficial for their health. “Poor preoperative health” and “lack of motivation and encouragement” were identified as the main barriers to the uptake of a preoperative online program, while program ‘simplicity’ and perceived ‘benefits’ were the main facilitators. Significant improvement in postoperative outcomes is perceived to influence patients’ willingness to participate in a preoperative multimodal e-Health program. Gastrointestinal cancer patients perceived the adoption of a preoperative multimodal e-Health application as safe to be performed at home and of potential benefit to their health. A range of patient’s characteristics, barriers and facilitators to the uptake of an online program were identified. These should be considered in future preoperative multimodal online programs to enhance patient experience, adherence and efficacy. The safety and efficacy of the online prehabilitation program will need to be determined in a larger randomized controlled trial.

## 1. Introduction

In 2020, approximately 20 million new cases of cancer were diagnosed worldwide, and almost 10 million cancer-related deaths were reported [1]. For selected patients presenting with advanced primary or locally recurrent cancer within the pelvic and abdominal regions, surgery with clear resection margin is considered the main curative treatment option, with 40% to 70% surviving at least 5 years after surgery [2,3]. Despite the drastically improved survival rates demonstrated over the last two decades, high rates of postoperative complications remain common, resulting in an increased length of hospital stay, reduced quality of life and increased health service costs [4]. This has been observed especially in cancer patients with lower physical activity levels [5,6], functional capacity [7,8], psychological status [9,10], frailty [11], patients who are malnourished [12,13] during the preoperative period and patients requiring neoadjuvant therapy in the weeks prior to surgery [7]. This directly contributes to the burden of healthcare costs attributed to these patients [14,15,16]. Therefore, there is a need to optimise surgical cancer patients to reduce the risk of postoperative complications.

To overcome this, several randomised controlled trials have been published in the last few years, investigating the effectiveness of a wide range of preoperative interventions on postoperative surgical outcomes for patients with gastrointestinal cancer [17,18,19,20]. There is now strong evidence that preoperative multimodal interventions, including exercise, nutrition and psychological support, are effective in reducing the rate of postoperative complications and length of hospital stay [21,22]. Many of these preoperative programs are delivered face-to-face in centralised rehabilitation centres [23,24]. While this may be suitable for some patients, such arrangements cannot readily accommodate patients living in regional or remote areas; who are from a low socio-economic status; or who may be juggling full-time work, family responsibilities and medical appointments in the weeks prior to the surgery. Home-based exercise prescription may help, although poor exercise fidelity and poor adherence to unsupervised exercise programs are commonly reported in the literature [25,26]. Due to the limited preoperative workup period, ranging from 2–6 weeks, there is a need to improve adherence to preoperative interventions.

A potential solution to these limitations is the implementation of a preoperative multimodal intervention via an e-Health app, allowing access to an individualised intervention at home. Recently, a published systematic review explored the evidence of technology-driven preoperative interventions on postoperative complication rates, length of hospital stay and quality of life outcomes in patients undergoing cancer surgery [27]. Surprisingly, only a small number of randomised controlled trials were identified [27]. The restrictions imposed by the COVID-19 pandemic have accelerated the research development within digital health technologies [28]. Thus, there is a potential opportunity to contribute towards the evidence on preoperative interventions delivered via an App. A detailed assessment of the barriers and facilitators to the adoption of technology during the preoperative period in patients undergoing gastrointestinal cancer surgery is required prior to the codesign and development of a conclusive randomised controlled effectiveness trial. The main aim of this project therefore was to explore barriers, facilitators and adoption of a preoperative multimodal intervention delivery via an online app in patients undergoing gastrointestinal cancer surgery. This preliminary work would support the development of a larger randomized controlled trial investigating the safety and efficacy of the online prehabilitation program in patients undergoing cancer surgery.

## 2. Materials and Methods

### 2.1. Study Design and Setting

This cross-sectional study explored the adoption of a prehabilitation multimodal online program in patients undergoing gastrointestinal cancer surgery at the Royal Prince Alfred Hospital (RPAH) between October 2021 and November 2022. This design was selected as it is a quick and unexpensive design used to study the exposure (prehabilitation multimodal online program) and outcome (barriers, facilitators and adoption) at the same time [29]. A study-specific questionnaire was developed in conjunction with validated, self-reported questionnaires investigating physical activity and nutritional status. This manuscript followed the reporting recommendations from the STROBE checklist [30]. Ethics approval and governance authorisation were obtained from the Royal Prince Alfred Hospital Research Ethics and Governance Offices (X21-0275-2021/STE03065).

### 2.2. Participants

Patients aged 18–85 years recovering (in-hospital) from elective lower gastrointestinal cancer surgery for primary or recurrent malignancies were eligible to participate. Patients were excluded if they have cognitive impairment such that they are unable to give informed consent and/or inadequate English to complete the study survey or verbal instructions. Participants were identified by their treating team and provided with the patient information sheet and consent form. An experienced research officer confirmed eligibility and answered any questions. Consenting patients were provided with the prehabilitation multimodal e-Health program.

### 2.3. Preoperative Multimodal App

Consenting participants were provided with a demonstration of a prehabilitation multimodal e-Health program, delivered on a study iPad. The e-Health exercise program, called StandingTall [31,32], was originally designed to deliver unsupervised, personalised functional balance and strength exercises at home to older adults for falls prevention (detailed information of the program can be found here: https://www.standingtall.org.au/ accessed on 1 September 2023). For the purposes of this study, StandingTall incorporated gentle warm-up exercises, sit-to-stand exercises and stepping exercises that can be undertaken with weights, to improve exercise capacity and overall muscular fitness. These exercises were appropriate for our population and were used, together with the nutritional and psychological advice. Participants were instructed on how to use the program by an experienced research officer. The study iPad was left with the participants for a minimal of 24 h. Participants were instructed to navigate the preoperative multimodal online program during this period, but not to complete the exercises or follow the nutritional or psychological advice, as all patients were recovering from surgery in hospital. This population was selected as they would be able to better recall the preoperative period and provide greater insight into the developed online program.

### 2.4. Study Survey

Patient characteristics, experiences, barriers and facilitators related to the preoperative multimodal program were explored, aimed at guiding the future development of a standalone preoperative program. The developed survey was piloted in five patients, five clinicians and one cancer consumer. The feedback provided during the development process helped identify key areas of focus, rewording of questions and helped deciding on which questions should be retained or dropped. The following open-ended question was used to identification of barriers and facilitators related to the preoperative multimodal program, “Please describe what would be the main facilitators/barriers for the uptake of a preoperative online program involving exercise, nutrition and psychological intervention?”.

The length of time each participants spent navigating the exercises was not recorded, but participants were instructed to navigate the program as much as possible. All participants reported that they have explored all features of the online preoperative multimodal program.

Patient outcomes included their characteristics (age, gender, weight, height, marital status, country born, language, level of education, and employment status), self-reported nutritional (PG-SGA Short Form) [33], and physical activity (IPAQ-SF) [34], previous conditions, disease and symptoms, technology use, view on the preoperative online program, main facilitators and barriers to the preoperative program, and their willingness to complete a preoperative program based on a wide range of outcomes. All surveys were entered and stored in REDCap (Research Electronic Data Capture) [35].

### 2.5. Sample Size and Statistical Analysis

Due to the design of this study, we have used a non-probability sampling method [24], where we aimed to recruit 30 participants. Descriptive statistics were used to characterise the sample, with data presented as frequency (percentage). The barriers and facilitators to the uptake of a preoperative multimodal online program were analysed through thematic framework analysis including familiarisation and review of data, coding of initial responses, and definition of themes [36]. Two authors coded the responses and generated the final themes (DS and JB), with any disagreement resolved via discussion. Equal weight as given to all data to develop as many codes as possible. The study investigators sorted the codes into main themes.

## 3. Results

### 3.1. Characteristics of the Included Participants

During the study period, a total of 30 participants navigated the preoperative multimodal online program. Of these, most were female (80.0%), born in Australia (70.0%), with a university degree (56.7%). Overall, 80.0% of participants were engaged in moderate or high physical activities, 50.0% of patients were at high risk of malnutrition. Between 3.4% and 31.0% of the sample reported an adverse health condition. Detailed characteristics of the included participants are described in Table 1.

### 3.2. Technology Use

Most of the included participants owned an Apple or Android smartphone (96.7%), iPad or tablet (56.7%), and a computer or laptop (76.7%), with the majority using these devices at least once a day to check their emails and browse the internet. Most of the included participants were confident or extremely confident about the use of technology (e.g., mobile devices and computers). Table 2 provides a detail information on the use of technology across the included participants.

### 3.3. Views on Preoperative Multimodal App

The views of the included participants towards a preoperative online program are included in Table 3. Most of the participants agree or strongly agree that an online program would benefit their recovery, can be completed at home safely, the inclusion of preoperative exercise, nutritional and psychological support is of relevance to them, and think the overall design of the program was appropriate.

### 3.4. Facilitators and Barriers to a Preoperative Online Program

Of the 19 (63.3%) patients that responded to this section of the study survey, the main facilitators to the uptake of a preoperative online program included two main overarching themes related to “simplicity”, and “benefit”. “Simplicity” was related to the preoperative multimodal program being easy to use and understand, and “Benefit” was related to the perception of effectiveness of the program on improving their health and promote wellbeing.

Of the 16 (53.3%) patients that responded to this section of the study survey, the main barriers to the uptake of a preoperative online program included three main overarching themes related to “poor preoperative health”, “lack of motivation” and “lack of encouragement”. “Poor preoperative health” was related to the presence of comorbidities and symptoms (e.g., hypertension, pain and bowel function), “lack of motivation” was related to lack of enthusiasm, difficulty in getting started and lack of clear goals, while “lack of encouragement” was related to lack of support/involvement (e.g., treating team and family) to reinforce they were on the right track.

### 3.5. Minimum Expected Outcomes from Preoperative Online Program

The minimal effectiveness of the preoperative online program on postoperative outcomes expected by the participants are detailed in Table 4. Most of the participants would expect at least a 50.0% reduction in postoperative complications (68.0%), a reduction in length of hospital stays by 4 days or more (52.0%), a 50% improvement in postoperative quality of life (52.0%) and a50.0% reduction in postoperative pain (54.0%). Interestingly, a reduction in hospital costs seems not to influence the willingness of participants to uptake an online preoperative program, with 40.9% expecting $0 reduction in costs.

## 4. Discussion

This study describes the views and expectations of gastrointestinal cancer patients on the adoption of a preoperative multimodal intervention delivered via e-Health. We found that most patients undergoing surgery are confident using technology, perceived the preoperative online program safe to be delivered at home, and of potential benefit to their wellbeing. The safety and efficacy of the prehabilitation online program would require to be explored in a larger randomised controlled trial. “Poor preoperative health”, “lack of motivation and “lack of personal encouragement” were identified as the main barriers to the uptake of a preoperative online program. Whereas the “simplicity” of the program and the perceived “benefits” were identified as the main facilitators to the uptake of a preoperative online program. Clinically significant reductions on postoperative complications, length of hospital stay and pain, and improvement in quality-of-life outcomes are some of the outcomes perceived to influence patients’ willingness to participate in a preoperative multimodal online intervention.

This is one of the first studies to seek consumer feedback on the development of a preoperative multimodal intervention delivered via an e-Health app. The information gathered in this study will support the development and guidance of future preoperative online applications for patients undergoing cancer surgery. Evidence suggests that adherence to unsupervised programs that are delivered at home is poor when compared to face-to-face programs [37]. This has detrimental effects on patients’ well-being and clinical outcomes. Interestingly, the results of this study highlight some of the reasons that were previously associated with poor adherence to a home exercise rehabilitation in musculoskeletal populations [38]. This included factors such as clear benefit of the intervention, presence of comorbidities and low support. On the other hand, the implementation of an e-Health program, in comparison to unsupervised home programs, is associated with increased adherence, especially in the short term [39].

Other studies are currently developing mobile apps as a tool to facilitate the delivery of prehabilitation. Wang et al., 2021 described a mobile app including a prehabilitation program for elective patients (N = 8 participants) [40]. This included preoperative exercise, nutrition and psychological support. The overall users’ satisfaction and usability of the mobile app was rated as high (e.g., score 4.5 out 5). Similarly, in our study the “simplicity” of the online program was associated with improved compliance and adherence. Interestingly, another factor identified in a preoperative mHealth app for patients undergoing major surgery was the individualization of the program [41]. In our study, this was not mentioned by the included participants. One of the main reasons may be due to the design of our online multimodal prehabilitation program, where participants’ score each session (e.g., easy to challenge), moderating the consequent sessions (e.g., participants that rated “easy” would have their next session more challenging). This allows for the intervention to be tailored to each individual participant. The study conducted by Kadiri et al. 2019 tested the feasibility of a home-based app in patients undergoing lung surgery [42]. Patients using the app (N = 31) were able to undergo four times more sessions than patients attending face-to-face sessions (N = 34). This demonstrate the utility of the prehabilitation online program, especially for patients undergoing cancer surgery, where the work up period is approximately 4–6 weeks. “Lack of motivation” was one of the main barriers to the uptake of prehabilitation reported in the current study. Higher levels of motivation were associated with increased adherence in a study using a mobile app as a support for pelvic floor muscle exercise training before prostate cancer surgery [43]. One of the key features of their app includes reminder functions and features that motivate users. These features should be considered during the development of prehabilitation online programs.

Despite most of the participants reporting no preoperative comorbidities and being confident on the use of technology, some participants reported the presence of multiple comorbidities and lack of confidence when using mobile devices. This information is important for the development of future preoperative e-Health programs. Previous studies have reported an increased number of comorbidities within cancer patients, and an association with greater risk of postoperative complications [44]. Thus, it is critical that a multidisciplinary team assesses all patients during the preoperative period to identify any potential comorbidities that may pose increased postoperative risks to patients. While poor preoperative health was identified as a barrier to the uptake of the preoperative e-Health program, it may also influence patient enrolment, yet patients with high comorbidity are the ones that would benefit most from a prehabilitation program. There is a need to ensure cancer patients are educated about the presence of comorbidity and the benefit of prehabilitation in preventing postoperative morbidity in this population. Historically, the adoption of technology across older adults is low, however, this is steadily increasing [45]. To enhance participant experience and adherence to preoperative online programs, it is essential that appropriate support is available to less technology savvy patients. In addition, approximately 5% of the study cohort do not own a smartphone, and 40% do not own a tablet or computer. Therefore, future studies should consider this in their budget, to make sure these patients are included.

One of the main barriers to the uptake of a preoperative multimodal e-Health program included “poor preoperative health”. Presence of preoperative comorbidities are common amongst cancer patients undergoing surgery. In our study, high or low blood pressure were present in 40% of the sample. It is recommended that patients presenting with these comorbidities are treated appropriately, so they can undertake the preoperative multimodal online program safely. Hearing and visual impairments were observed in over 30% of the patients. Preoperative online programs should be modifiable to patient’s needs, and the ability to easily adjust display light, font and volume is required for this population. Other barriers identified included, “lack of motivation” and “lack of encouragement”. Online motivational coaching, incorporating combined strategies of supervision, feedback, and reinforcement has been suggested to increase adherence to exercise programs [38]. Interestingly, strategies to support behaviour change and increase motivation in an e-Health program were deemed not necessary in community-dwelling older people [46]. The inclusion of personal encouragement, with health professional check-ins could, also enhanced encouragement.

Facilitators to the uptake of this preoperative multimodal e-Health program are that is easy to use and navigate and provides information on the benefits of patients’ wellbeing. Interestingly, whereas most patients would accept an approximately 50% reduction on postoperative complications and a reduction of 3 days on the length of hospital stay as the minimal outcomes perceived to influence their willingness to participate in a preoperative multimodal online intervention, a considerable number of patients indicated higher postoperative improvements, which are currently not supported by the prehabilitation literature [47]. Clear evidence-based information on the benefits of prehabilitation during the preoperative period would enhance patient expectations and experience.

One of the strengths of this study is the inclusion of participants with lived experiences of gastrointestinal cancer. The recruitment was completed during their postoperative in-hospital recovery period, following gastrointestinal cancer surgery, thus facilitating recall of the preoperative period (i.e., time of which the preoperative multimodal program would be applied). In addition, this study includes a range of questions that provided greater details of their characteristics and perspectives towards the adoption of a preoperative multimodal online program. The main limitations are the bias toward the highly educated, predominantly female population, who were comfortable using technology. Additionally, patients were recruited from a single tertiary hospital following gastrointestinal surgery. Thus, it is important to note that the results of this study may not be generalised to other settings and populations. Furthermore, only 50–60% of the participants responded to the questions related to facilitators and barriers to a preoperative online program. This may be because they did not actually try the exercise program. Future studies investigating different consumer aspects and perspectives are warranted to further support the development and guidance of preoperative multimodal online programs.

Prehabilitation e-Health programs have the potential to improve preoperative physical, nutritional and psychological outcomes of patients undergoing cancer surgery, leading to a reduced number of postoperative complications and shorter recovery time. This technology can be used for delivering a prehabilitation program for patients undergoing surgery in the comfort of their homes, especially for patients that live in rural or remote areas. Future prehabilitation trials should consider the information reported in this study to further support the development of e-Health programs for patients undergoing cancer surgery.

## 5. Conclusions

Gastrointestinal cancer patients perceived the preoperative multimodal e-health application safe to be performed at home and of potential benefit to their health. Several patient’s expectations, barriers and facilitators were identified. These should be considered in future preoperative multimodal online programs to enhance patient experience, adherence and efficacy. The safety and efficacy of the online prehabilitation program should be tested in an appropriately powered randomized controlled trial.

## Figures and Tables

**Table 1:** Characteristics of the included sample (N = 30).

Variables	Estimates
Age, Years	54 [23 to 95]
Gender, Female	24 [80.0%]
Relationship	
Single/Divorced/Widowed	20 [66.7%]
Married/Living with a partner	10 [33.3%]
Country of Birth, Australia	21 [70.0%]
Language, English	27 [90.0%]
Caring responsibilities	10 [33.3%]
Education	
None–Year 12	9 [30.0%]
Technical Certificate or Diploma	4 [13.3%]
University Degree	17 [56.7%]
Employment Status	
Full-time/Part-time/Casual	15 [50.0%]
Retired/Sick Leave/Student/Unemployed	15 [50.0%]
Access to Paid Sick Leave *	11 [31.9%]
Weekly Personal Income ^#^	
$0	2 [7.1%]
$1–$399 ($1–$20,799)	6 [21.4%]
$400–$1249 ($20,800–$64,999)	10 [35.7%]
≥$1250 (≥$65,000)	10 [35.7%]
Risk of Malnutrition (PG-SGA Short Form)	
Low Risk (0–3 points)	6 [20.0%]
Medium Risk (4–8 points)	9 [30.0%]
High Risk (>9 points)	15 [50.0%]
Physical Activity (IPAQ-SF)	
High	10 [33.3%]
Moderate	14 [46.7%]
Low	6 [20.0%]
Health Conditions *	
Hearing Impairment	8 [27.6%]
Visual Impairment	9 [31.0%]
Peripheral vascular disease	2 [6.9%]
Diabetes	4 [13.3%]
Heart Disease	1 [3.4%]
High Blood Pressure	6 [20.0%]
Low Blood Pressure	4 [13.8%]
High Cholesterol	3 [10.3%]
Incontinence	6 [20.7%]
Chronic Lung Disease	1 [3.4%]
Bodily Pain	
None	2 [6.7%]
Mild/Very Mild	9 [30.0%]
Moderate/Severe/Very Severe	19 [63.3%]
Pain Interfere with normal work	
None	5 [16.7%]
Mild/Very Mild	7 [23.3%]
Moderate/Severe/Very Severe	18 [60.0%]

Data presented as frequency (percentage) or median (interquartile range); * N = 29; ^#^ N = 28.

**Table 2:** Use of technology across the included participants (N = 30).

Variables						Estimates				
Own Apple iPhone/Android Smartphone						29 [96.7%]				
Own Apple iPad/Android Tablet						17 [56.7%]				
Frequency of Apple iPad/Android Tablet use *										
More than once a day						10 [58.8%]				
About once a day						4 [23.5%]				
More than once a week						1 [5.9%]				
More than once a month						1 [5.9%]				
Less than once a month						1 [5.9%]				
Use of Apple iPad/Android Tablet *										
Viewing Pictures						7 [41.2%]				
Email						12 [70.6%]				
Playing games						7 [41.2%]				
Internet (Checking facts)						10 [58.8%]				
Internet (Social networking, e.g., Facebook)						11 [64.7%]				
Internet (Banking)						13 [76.5%]				
Internet (Purchasing)						11 [64.7%]				
Other						5 [29.4%]				
Own Computer/Laptop						23 [76.7%]				
Access to Computer/Laptop						26 [86.7%]				
Used a Computer/Laptop						29 [96.7%]				
Frequency of Computer/Laptop use *^#^*										
More than once a day						11 [47.8%]				
About once a day						8 [34.8%]				
More than once a week						3 [13.0%]				
Less than once a month						1 [4.3%]				
Use of Computer/Laptop^#^										
Word Processing						13 [56.5%]				
Email						19 [82.6%]				
Playing games						2 [8.7%]				
Internet (Checking facts)						15 [65.2%]				
Internet (Social networking, e.g., Facebook)						15 [65.2%]				
Internet (Banking)						19 [82.6%]				
Internet (Purchasing)						18 [78.3%]				
Database/Spreadsheets						10 [43.5%]				
Other						5 [21.7%]				
Confidence/ability using Technology ^										
0 (No confidence/ability)	1	2	3	4	5	6	7	8	9	10 (Extremely confident)
1 [3.6%]	0 [0.0%]	2 [7.1%]	1 [3.6%]	0 [0.0%]	2 [7.1%]	2 [7.1%]	5 [17.9%]	3 [10.7%]	4 [14.3%]	8 [28.6%]

Data presented as frequency (percentage). * N = 17; ^#^ N = 23; ^ N = 28.

**Table 3:** Views on preoperative online program (N = 29).

Questions	Disagree/Strongly Disagree	Neither Agree/Disagree	Agree/Strongly Agree
Doing the preoperative online program would be good for me	2 [6.9%]	5 [17.2%]	22 [75.9%]
Doing the preoperative online program would help with my recovery after the surgery	1 [3.4%]	5 [17.2%]	23 [79.3%]
Doing the preoperative online program would improve my overall health and wellbeing	1 [3.4%]	7 [24.1%]	21 [72.4%]
I would feel confident in doing the preoperative online program at home	2 [6.9%]	2 [6.9%]	25 [86.2%]
I would feel safe in doing the preoperative online program at home	1 [3.4%]	2 [6.9%]	26 [89.7%]
I would have enough time to do the preoperative online program	1 [3.4%]	6 [20.7%]	22 [75.9%]
I would have support from people whose opinions matter to me, to perform the preoperative exercise program	1 [3.4%]	4 [13.8%]	24 [82.8%]
I would feel confident in recommending the preoperative online program to other people	1 [3.4%]	5 [17.2%]	23 [79.3%]
The exercise program would be relevant to me *	3 [10.7%]	5 [17.9%]	20 [71.4%]
The nutritional information would be relevant to me	1 [3.4%]	5 [17.2%]	23 [79.3%]
The psychological information would be relevant to me *	3 [10.7%]	6 [21.4%]	19 [67.9%]
I would not be concerned about privacy when using the preoperative online program *	3 [10.7%]	5 [17.9%]	20 [71.4%]
I would require technical support to navigate the preoperative online program *	18 [64.3%]	4 [14.3%]	6 [21.4%]
I would do the preoperative online program if my surgeon recommends it	1 [3.4%]	1 [3.4%]	27 [93.1%]
The wording used in the online program was appropriate and easy to understand	1 [3.4%]	1 [3.4%]	27 [93.1%]
The length of the online program is appropriate ^	4 [16.0%]	6 [24.0%]	15 [60.0%]
The online program instructions are easy to understand ^	0 [0.0%]	2 [8.0%]	23 [92.0%]
The design of the online program is appropriate ^	1 [4.0%]	1 [4.0%]	23 [92.0%]
The scoring of the exercise program was appropriate ^	0 [0.0%]	5 [20.0%]	20 [80.0%]

Data presented as frequency (percentage); * N = 28; ^ N = 25.

**Table 4:** Minimum outcomes expected from preoperative online program (N = 25).

**Reduction in chances of acquiring a complication**										
**0%**	**10%**	**20%**	**30%**	**40%**	**50%**	**60%**	**70%**	**80%**	**90%**	**100%**
1 [4.0%]	5 [20.0%]	3 [12.0%]	0 [0.0%]	4 [16.0%]	4 [16.0%]	2 [8.0%]	1 [4.0%]	3 [12.0%]	1 [4.0%]	1 [4.0%]
**Reduction in length of hospital stay**										
**0 day**	**1 day**	**2 days**	**3 days**	**4 days**	**5 days**	**6 days**	**7 days**	**8 days**	**9 days**	**10 days**
1 [4.0%]	4 [16.0%]	3 [12.0%]	5 [20.0%]	2 [8.0%]	1 [4.0%]	0 [0.0%]	6 [24.0%]	1 [4.0%]	0 [0.0%]	2 [8.0%]
**Improvement in postoperative quality of life**										
**0%**	**10%**	**20%**	**30%**	**40%**	**50%**	**60%**	**70%**	**80%**	**90%**	**100%**
2 [8.0%]	3 [12.0%]	3 [12.0%]	1 [4.0%]	2 [8.0%]	2 [8.0%]	2 [8.0%]	2 [8.0%]	3 [12.0%]	1 [4.0%]	4 [16.0%]
Reduction in postoperative pain *										
**0%**	**10%**	**20%**	**30%**	**40%**	**50%**	**60%**	**70%**	**80%**	**90%**	**100%**
0 [0.0%]	4 [15.4%]	5 [19.2%]	1 [3.8%]	1 [3.8%]	3 [11.5%]	2 [7.7%]	2 [7.7%]	3 [11.5%]	2 [7.7%]	3 [11.5%]
**Reduction in hospital costs ^**										
**$0**	**$1000**		**$3000**		**$5000**	**$10,000**	**$15,000**	**$20,000**	**$30,000**	**$50,000**
9 [40.9%]	3 [13.6%]		2 [9.1%]		1 [4.5%]	1 [4.5%]	0 [0.0%]	2 [9.1%]	1 [4.5%]	3 [13.6%]

Data presented as frequency (percentage); * N = 26; ^ N = 22.

## Data Availability

Data is available through reasonable request to the corresponding author.
